# Data Integration Challenges for Machine Learning in Precision Medicine

**DOI:** 10.3389/fmed.2021.784455

**Published:** 2022-01-25

**Authors:** Mireya Martínez-García, Enrique Hernández-Lemus

**Affiliations:** ^1^Clinical Research Division, National Institute of Cardiology ‘Ignacio Chávez’, Mexico City, Mexico; ^2^Computational Genomics Division, National Institute of Genomic Medicine (INMEGEN), Mexico City, Mexico; ^3^Center for Complexity Sciences, Universidad Nacional Autnoma de Mexico, Mexico City, Mexico

**Keywords:** precision medicine, machine learning, data integration, meta-data mining, computational intelligence

## Abstract

A main goal of Precision Medicine is that of incorporating and integrating the vast corpora on different databases about the molecular and environmental origins of disease, into analytic frameworks, allowing the development of individualized, context-dependent diagnostics, and therapeutic approaches. In this regard, artificial intelligence and machine learning approaches can be used to build analytical models of complex disease aimed at prediction of personalized health conditions and outcomes. Such models must handle the wide heterogeneity of individuals in both their genetic predisposition and their social and environmental determinants. Computational approaches to medicine need to be able to efficiently manage, visualize and integrate, large datasets combining structure, and unstructured formats. This needs to be done while constrained by different levels of confidentiality, ideally doing so within a unified analytical architecture. Efficient data integration and management is key to the successful application of computational intelligence approaches to medicine. A number of challenges arise in the design of successful designs to medical data analytics under currently demanding conditions of performance in personalized medicine, while also subject to time, computational power, and bioethical constraints. Here, we will review some of these constraints and discuss possible avenues to overcome current challenges.

## 1. Introduction

Contemporary biomedical research and medical practices are increasingly turning into data-intensive fields, for which computational intelligence approaches, such as those based on artificial intelligence and machine learning (AI/ML) methods are becoming the norm. Due to the specific nature of these fields, the integration and management of the ever-growing volumes of heterogeneous data involved, often presents a number of challenges. These challenges become even more relevant in the light of the importance that AI/ML are gaining, establishing themselves at the core of the state-of-the-art in biomedical research and clinical medicine (1–3), as well as public health and healthcare policy (4–6).

From the standpoint of biomedical research, a number of large, data-intensive collaborative projects, such as the International Hap Map project (7, 8), The Cancer Genome Atlas (TCGA) (9–12), the 1000 Genomes (1000G) study (13–16), the GTEX consortium (17–19), and the Human Cell Atlas (HCA) (20, 21), and others are establishing novel frameworks for the molecular study of health and disease. Such frameworks are firmly supported by robust database management and integration strategies that are allowing them to develop into central tools for basic and translational biomedical research.

Relevant as genomics and high throughput molecular studies are for biomedicine, there are other relevant players in the medical data arena. Among the more important in the present context are large scale clinical and phenotypic studies. Large clinical cohorts creating data-intensive outputs are of course not new, but the extent of their outreach and the complexity of the resulting data sets are growing exponentially fast. Starting from large scale clinical surveys, such as the Framingham Heart study (22, 23), the Wellcome Trust Case Control Consortium (24) and moving unto efforts like the UK Biobank that combines large scale clinic and phenotypic data with ultra-high-throughput genomic testing (25–28) that for the last 15 years has been generating massive data corpora used for their own means but also encouraging and feeding other data-intensive analytical efforts from genetic disease association (29) to brain imaging (30) to psychology (31) and social determinants of health (32), to name just a few instances. It goes without saying that the impact that these projects have reached on the basic and clinical settings, but also in the epidemiology and public health areas has been enormous.

In the context of AI/ML, however, the focus is shifting into *translating* the astronomical amounts of data generated ultimately into *products* and *policies* able to impact both the patients' and the general public health. This has been, for instance, one of the central goals of the U.S. initiative in Personalized Medicine (33, 34). That is, how to develop analytic strategies—many of them founded on automated learning, essential, given the size of complexities of current health-related data corpora—to pass from large scale, heterogeneous data to useful (even actionable) medical information (35).

Aside from large scale, even multi-national efforts—such as the ones in the consortia just discussed—, another area of intensive interest regarding data-mining in medicine has been the development of analytical strategies to effectively mine the ever growing body of Electronic Health Records (EHR), that has been perceived as a largely forgone and under-utilized data source (6, 36–39).

One main challenge in knowledge discovery from EHRs is that electronic medical records are highly heterogeneous data sources with a complex array of quantitative, qualitative, and transactional data. Disparate data types include ICD codes (mainly used for pricing and charging hospital procedures), biochemical and lab tests, clinical (text-based) notes, historical archives of medical interventions, therapies and even pharmaceutical deliveries. These data sources are often captured by dozens of individuals (sometimes with biased criteria) for each instance. Hence EHR data is quite difficult to analyze, in particular if one is looking (as is often the case if AI/ML techniques are being considered) multi-patient institutional and even multi-centric levels.

In brief, EHRs were not developed to be used as a resource for automated learning so they are not designed with *data structures* in mind. Since EHRs are first and foremost adapted for clinical and hospital logistics, data modeling and learning will often face challenges related to structural heterogeneity from their early stages, either by adapting existing EHR strategies or by re-designing them (40–44).

In the quest for more efficient healthcare interventions, based on information-optimized clinical practice and policy, AI/ML will certainly play a key role in going from a medicine approach—based mainly in the skills of the well-trained clinician—to one based also in detailed (often automated) analysis of the individualized interplay of molecular interactions and physiological traits with environmental and even social elements, thus, delivering the promise of personalized medicine (1, 2, 45, 46). The development of this analytic approach to personalized medicine (often termed *Precision Medicine*) involves a number of theoretical frameworks from systems biology to computational biology, biomedical informatics, and computational medicine. This is so, since health and healthcare are multi-dimensional in nature, hence, their study must consider information at the genetic, molecular, clinical and population levels. Health and healthcare analytics, however, must also evaluate and assess how to cope with the complexity and natural biases of the plethora of medical-related databases in which said molecular, clinical, and epidemiological data resides. This, again, points out to the need of customized, scalable computational and analytical tools for pattern discovery and hypothesis generation and testing. AI/ML is turning into a cornerstone of personalized medicine (6, 47–49).

In order to present a panoramic view on how these and other challenges may be overcome toward an optimized application of machine learning and artificial intelligence to analyze biomedical and health-related data in a Precision Medicine context, the rest of this work will proceed as follows: The next section (The role of data in training good AI/ML models) will establish the necessity to have proper data as input to machine learning and AI models useful in Precision Medicine. We will discuss how having very large data corpora (a.k.a *Big Data*) is great, but often carries with it the so-called *curse of dimensionality* and the need to perform *feature selection*, i.e., to select relevant pieces of information among very large and complex databases. We will also elaborate on the challenges created by diverse and heterogeneous data types and sources, bringing problems, such as *class imbalance* (study groups of sometimes extremely disparate sizes, that are problematic to analyze for many machine learning algorithms).

The following section (Precision medicine: transforming biomedical evidence with data analytics) will outline how the tenets of computational intelligence and machine learning may be used to advance medicine turning it (even more) into a full-evidence based science. We will see that in order to impact biomedical research, clinical practice and public policy, AI/ML approaches could be helpful to extend our capacities to generate biomedical knowledge, contribute to knowledge dissemination, translate personalized medicine into clinical practice and even empowering the patients. In order to develop, large scale data analytics in medicine should be able to become *translational*, i.e., moving faster from research environments to clinical settings to ultimately benefit the patients. Then, we will move on in the next section, to discuss the main challenges involved in the use of computational learning toward Precision Medicine. These include processing heterogeneous and unstructured data, working on collaborative and cloud-based resources, developing standards for data sharing and collaboration, implementing software solutions to support large scale data analytics under the biomedical and clinical diverse data ecosystems.

Section 5 will deal with one of the main challenges involved in the quest to effectively implement AI/ML in Precision Medicine: Data Integration. Biomedical and clinical knowledge deals with a plethora of phenomena, ranging from the molecular to the socio-political. Currently, we have technologies to massively measure or infer data from most of these domains. How to *make sense* of these different dimensions to turn them into a coherent, intelligible body of knowledge useful for the researchers, but more importantly, for practising clinicians, the healthcare providers and the patients is an extremely challenging endeavor. Interestingly, a source of information that is becoming key for AI/ML approaches in Precision Medicine is metadata. Metadata, i.e., auxiliary data sources often used to define other data types. Having one's genome sequence is of little use if we do not have a proper *annotation* file; and knowledge of the zip code or educational level of a patient may provide actual clues for their *personalized* treatment. Since many data types are actually pre-processed prior to the analysis, it is also relevant to know how has the data been treated prior to its current form. Information of this kind is also considered metadata. Metadata is, hence, becoming more and more relevant. Managing such large amounts of *personal* data (what can be more personal for us than our healthcare data?), however, does not come without a price. Ethical and legal considerations pose no small problem if one is to provide fair and *minimally invasive* use of the data, especially if it is of a sensible or private nature. Some of these issues are discussed in section 6. Section 7 is devoted to present the Data Management Plan, a document that will be extremely useful to set the guidelines of any data-intensive project being a research protocol, a clinical trail or a healthcare management design. Finally, in section 8, we present some Conclusions and Perspectives.

## 2. The Role of Data in Training Good AI/ML Models

The current development of highly sophisticated and often quite effective AI/ML and the accompanying proliferation of large scale data sources in the biomedical setting, has raised the expectations regarding the many potential benefits that can be derived from the marriage of *good methods* + *good data*. However, in order for these large amounts of data to be useful in producing good AI/ML models, size is not the only thing that matters, a question that is often overlooked (50, 51).

**Figure 1:**
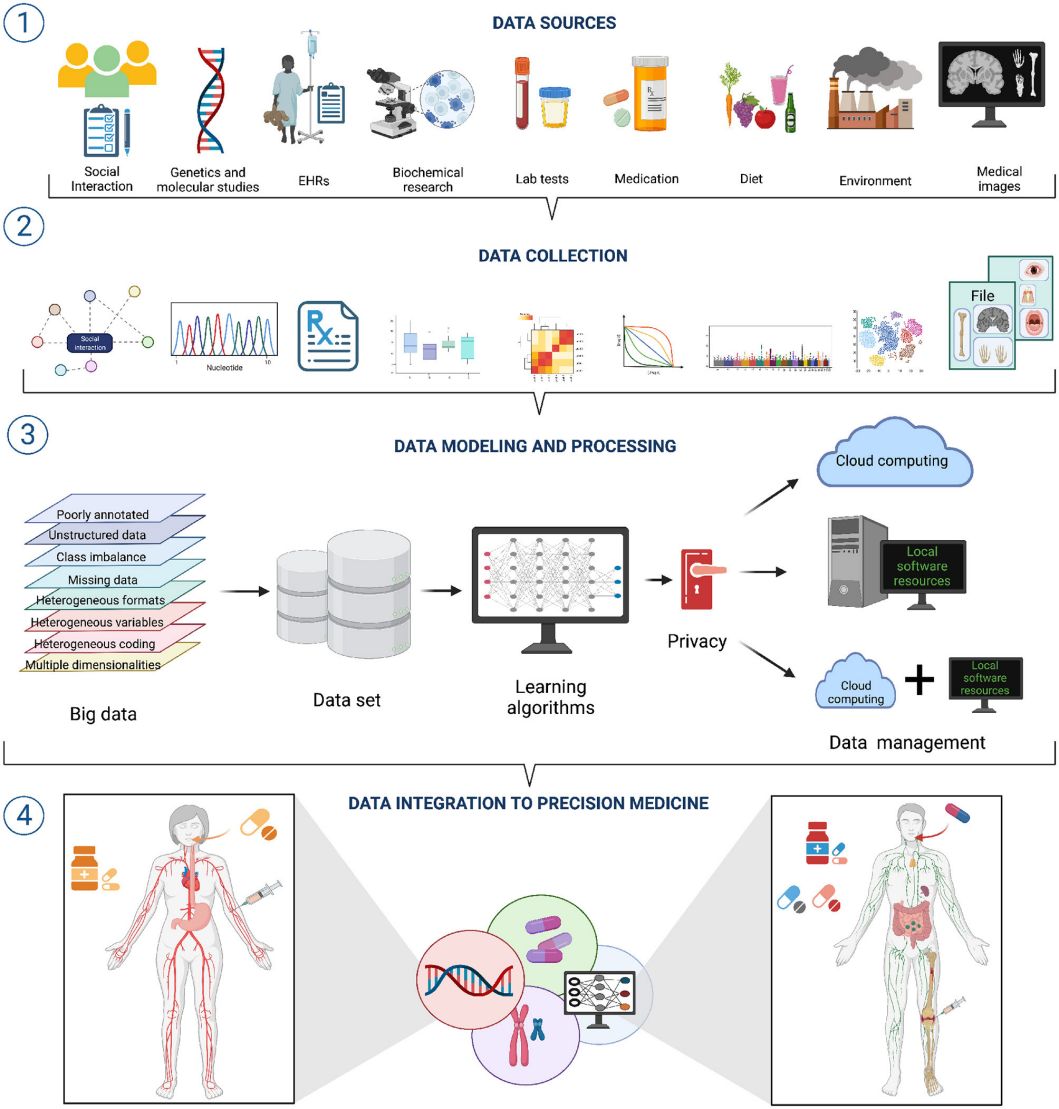
A workflow for data integration for AI/ML modeling in precision medicine. ① A wide variety of data sources with diverse features exists. Hence, different approaches to data collection and pre-processing are needed ②. ③ Integrating such diverse and heterogeneous data is one of the grand challenges to the successful application of AI/ML approaches to Precision Medicine. Overcoming such challenges will bring important improvements to Precision Medicine ④.

Clinical and biomedical data comes in a wide variety of sizes, forms, and formats; it is often complex, heterogeneous, poorly annotated, and often unstructured. Now, each of these issues: size, variety, formatting, complexity, heterogeneity, bad annotation, and lack of structure, pose a challenge to effective AI/ML modeling (see Figure 1 section ①) (52).

Regarding size, for instance, even when we often deal with *big data*—usually considered an advantage—, it is common that these data sources suffer from the so-called *curse of dimensionality* (CoD), a situation in which the number of variables or features is much larger than the number of experimental samples or realizations. CoD is particularly evident in the case of genomic and transcriptomic analyses for which the number of genes or transcripts is in the order of tens of thousands whereas the number of samples is rarely larger than a few hundreds or a few thousands at most. Even more complex is the scenario when one is measuring, for instance, chemical modifications, such as DNA methylation; the current experimental protocols allow for the simultaneous measures of five thousand hundred or more methylation probes (52).

CoD leads to the *p* > > *n* problem in machine learning (53): increased data dimensionality may cause AI/ML methods to suffer from **overfitting**. Overfitting, in turn, implies that the methods are highly accurate on training data while showing low performance on generalization or handling unseen data. Potentially good methods will fail to deliver in real life applications. One approach to deal with the CoD is performing data dimensionality reduction prior to training the ML methods. The most common means of data dimensionality reduction are *feature extraction* in which data is projected from a high dimensional space to a lower dimensional space and *feature selection* that reduces dimensions by identifying a relevant or *informative* subset of the original set of features (54).

Feature extraction methods, such as principal component analysis (PCA) and other methods based on eigenvalue decompositions, non-negative matrix factorization (NNF), *t*-distributed stochastic neighbor embedding (t-SNE) and others, allow for easier data visualization, exploration, and compression, as well as latent factor profiling. On the other hand, feature selection methods consists in one or more of the following strategies: data filtering (DF), data wrapping (DW), and data embedding (DE). The purpose the former (DF) is to select a subset of relevant features in a model independent fashion an include methodological approaches, such as ANOVA, Pearson's correlation, information theoretical measures, such as entropy and mutual information, constrained regressions, and maximal relevance minimal redundancy (mRMR) methods. DW methods look for the best combination of features trained by a particular predictive model and include the recursive feature elimination (RFE), jackstraw and the Boruta-Random Forests (BRF). DE are a combination of DF and DW that works by performing feature selection while building a predictive model, perhaps the best known example of DE method is the least absolute shrinkage and selection operator (LASSO) and its extensions, such as the elastic net algorithm (52).

Data variety/diversity and data heterogeneity also result problematic for the implementation of AI/ML modeling in Precision Medicine. Heterogeneity emerges from many situations, such as substantially different types of variables (or different coding) in the various data sets (think of EHRs from different hospitals), mismatched distributions or scaling including disparate dynamic ranges (say we have combined expression data from microarrays and RNASeq technologies), diverse data modalities (continuous signals, counts, intervals, categories, pathways, etc., derived from molecular and imaging experiments) and formats (say European versus American reporting standards) (Figure 1 section ②). Integrating heterogeneous data types may be done naively, by just concatenating features from disparate data sources, but this reduce the number of working to the use of decision tree (DT)—like models that suffer from overfitting. An alternative would be to use penalized regression (e.g., elastic nets) with several regularization strategies, though this may in turn bring challenges regarding interpretability of results (51, 52). Better results may be obtained by resorting to block-scaling (55) or multiple kernel learning methods (56).

Due to the complexity intrinsically associated to biomedical and clinical data, but also due to difficulties in subject/sample procuration and in data acquisition (data generating/sampling technologies may fail) it is common to have problematic circumstances, such as missing data (from instances not measured or measured defectively), class imbalance (widely different sample sizes in different feature groups) and even rarity (an extreme form of class imbalance) (57). There are several learning strategies to cope with missing data and class imbalance, ranging from the so-called *listwise deletion* (i.e., completely deleting the problematic sample from the study), imputation (i.e., inferring the missing value from expectation methods from the sample-wise profiles or even from feature-wise profiles) suing methods, such as *k*-nearest neighbor replacement, full conditional specification, stochastic gradient boosted trees, and other ensemble regression frameworks (52).

Class imbalance is another problematic-yet-pervasive situation in large scale data analytics (LSDA) of biomedical and clinical data. This fact becomes quite relevant since the most machine learning methods, such as support vector machines, random forests, and artificial neural networks assume balanced class distributions. Hence, these classifiers tend to overestimate patterns from the majority class, and underestimate those features characteristic of the minority class or classes. To overcome this limitation a class of ML approaches termed class imbalance learning (CIL) methods have been developed. CIL algorithms can be based on *data sampling* (e.g., random undersampling, bootstrap sampling, etc.); on *algorithm modifications* incorporating the inherent biases or skewness in the learning steps (e.g., weightedSVM, weigthedELM) or in *ensemble learning* in which several ML methods are applied and the results are consensed or averaged (52, 58).

Furthermore, even if most of these problematic issues may be solved, at least partially, with the analytic approaches just discussed, two relevant issues remain. First, real life datasets often have not one, but several (even all) of these challenging features. The ML methods useful to tackle some of these limitations may have poor performance due to others. Leveraging alternatives by evaluating the pros and cons may not be trivial. Second, every one of the methods for LSDA in imperfect/real-life datasets has its own set of assumptions and limitations. AI/ML researchers in biomedicine should be very aware of this and very cautious when combining methods and taking conclusions. However, as we will see in the next section, advancing biomedical and clinical research by using AI/ML approaches often worth all the efforts.

## 3. Precision Medicine: Transforming Biomedical Evidence With Data Analytics

Since the later years of the 20th century, following the pioneering work by Cochrane, Eddy, and others (59–62) efforts have been directed toward building a systematic approach to medical and public health decisions, one founded not on anechdotic or individual expertise, but rather in the light of a full inspection of the existing clinical and biomedical research. This approach, called Evidence-Based Medicine (EBM) (63) aimed at the comprehensive use of all the accumulated scientific and clinical evidence to develop health related interventions and policy. At that time EBM was founded on anecdotal clinical experience, published case reports, meta-analyses and systematic reviews, and randomized controlled trials (64, 65). No *high-throughput* molecular or individual disaggregated information was considered at the time; even the already existing large-scale epidemiological data was not exploited fully due to data availability constraints (66, 67).

Even if the EBM paradigm has been superseded for various reasons, perhaps its main relevance resided in bringing to attention the fact that, as a rule, healthcare-related decisions should be supported by objective, stringent evidence rather than being left to the subjective opinion of some *individual* professional, expert as they may be. With the advent of larger, well-curated data corpora and more powerful ways to analyze the data and transforming it into useful information, EBM ideals have been embraced and incorporated into what has been called Precision Medicine (68–71).

Aside from the spectacular changes in information technologies in recent times, another main booster of this transformation was the *genomic revolution* driven by the human genome project (HGP) (72–74). The promises of the HGP,—many of them still undelivered (75)—pointed out to data-based biomedicine (particularly the identification of genetic variants behind the diseased phenotypes), as a key player to identify targets and customize pharmacological and other therapeutic interventions leading to a dramatic improvement of population and individual health (76, 77).

In view of this emerging paradigm, what is the role that AI/ML may play in its establishment as the standard approach in biomedical research, clinical practice and public policy? It has been argued (2, 6, 78) that there are at least four development avenues in which LSDA may impact healthcare: (i) LSDA may enlarge the capacity to generate new biomedical knowledge, (ii) LSDA may provide a support for healthcare-related knowledge dissemination, (iii) LSDA can become a tool for translating personalized medicine initiatives into clinical practice (for instance, by integrating molecular and EHR data on a single framework), and (iv) LSDA supplemented with simplified user interfaces can become a vehicle for empowering of the patients, helping them play a more active role in their own healthcare decision making.

In order to deliver such benefits, LSDA needs to be able to address questions, such as how to deal with highly unstructured heterogeneous data (say from EHRs) via high-performance computational techniques for quantitative analytics, but also for data mining, literature mining, and natural language processing algorithms over integrated pipelines. Particularly challenging are the scenarios related to clinical practice since they would be ideally processing such enormous amounts of unstructured data in cuasi-real time, if LSDA is intended to be beneficial for the individual patient (79, 80). In the following sections, we will discuss some of the opportunities and limitations of applying AI/ML (often in the form of LSDA) in health-related settings.

### 3.1. Personalized Medicine: From Data Lakes to Patient Beds

LSDA and AI/ML may also play a role in supporting the clinical practitioners to keep up-to-date with the current scientific literature in their fields, an issue that has been struggling attending physicians for a while. In brief, if a medical doctor wants to treat their patients with the current best available therapeutic options, difficulties arise in trying to define what is *currently* considered better. As is known, the available scientific literature regarding a single medical speciality has been already overwhelming. The situation becomes much worse when one is dealing with multi-morbid patients since clinical guidelines and algorithms are often aimed at the single condition scenario (81–85).

Embracing the computational learning paradigm, the clinician may be armed with a new set of tools allowing for suggestions/surveys supported by real-time patient data analytics integrating, both the complexity of the patient's genetic background, environmental conditions, and the corresponding comorbidities with the current literature standards of care (Figure 1 section ③) (6, 33, 46, 86–88).

Aside from standard biomedical and clinical data, LSDA allows to further integrate occupational, social, physiological, and even behavioral information of the individual patient (available in social network, wearable devices, and other cloud-based resources) (89–92) to enhance the clinical profiles. To reach this point, however, there are important conundrums to be solved. In particular, novel computing and analytical frameworks should be designed to find patients' similarities and differences, but also to discover patterns highlighting their connections and discrepancies with the aim of calculating, for instance, personalized disease risk profiles, akin to polygenic risk scores, but under a much more general view—engulfing all the already discussed data types—allowing for individualized proactive medicine (93–95).

Hence, by integrating phenotype and disease-history based approaches, LSDA aims to advance personalized disease prediction, improve healthcare management and even contribute to an overall positive impact to individual wellness (Figure 1 section ④) (96–100). In doing so, AI/ML approaches are collaborating to a shift in the emphasis of clinical medicine from a disease-centered view to a patient-based practice (101, 102), a paradigm that has been long known since Hippocratic times and has been resumed a hundred years ago by the Spanish endocrinologist Gregorio Marañón who stated that *there are no diseases but patients*.

The panorama we have just discussed seem to be quite promising, indeed AI/ML and LSDA have already brought relevant advances toward Personalized Medicine (34, 70, 103). However, a consensus has not been reached as to how to integrate the large scale data of EHR, the many heterogeneous databases on molecular, phenotypical and environmental information derived from large scale experimental, clinical and epidemiologic studies and the individual-wise data gathered from disparate sources, such as social networks and wearable devices to develop a personalized approach to medicine? (46, 48, 104–106).

## 4. Challenges to Computational Learning in Precision Medicine

Of the many challenges posed to AI/ML by ever-growing health and biomedicine data sources, one of them is paradoxically related to what is often perceived as its main driving force. Having large amounts of data is obviously beneficial for computational learning algorithms, the more data you have, the more robust your classifiers, regressions, and mining strategies will be. However, as the tendencies move toward Precision Medicine, we can see how some major sources of primary biomedical information, such as genomics (in particular next generation sequencing) and imaging are becoming progressively cheaper (107–109), hence allowing their widespread use, nevertheless the computational costs of processing and analyzing the data are, for obvious reasons, growing fast (110–114).

Hence, aside from the already discussed challenges of database structural heterogeneity and data type integration, a number of major limitations for the development of AI/ML in biomedicine belong to the computer systems domain (115). Those challenges are, for instance, in the development of consolidation, characterization, validation, and processing standards for the data; creating ontologies and knowledge relationships for entities, such as genes, drugs, diseases, symptoms, patients, and treatments, as well as their corresponding entity-relationship schemes (116–119).

Along these lines, recent advances in AI, in particular those directed to Natural Language Processing (NLP) have been incorporating tools of semantic web analysis, such as conceptual relational networks (120, 121), semantic-syntactic classification (122), and similarity mapping (123). The problem, again, is a matter of throughput: effective implementation (training, in particular) of such NLP tools is only enabled if one has extremely large data corpora being accessed on a concurrent fashion (124). The vast majority of hospitals, research labs and even pharmaceutical development facilities do not currently have access to the storage and computational power resources needed to perform these analyses. The current alternative to local processing is, of course, cloud computing (125–127). However, as we will see in the next subsection, performing LSDA in medical and biomedical data in the cloud is not a problem-free solution.

### 4.1. Precision Medicine, Machine Learning and Cloud Computing

The use of cloud computing in the analysis of clinical, biomedical and healthcare data has many advantages: (i) it helps to solve the issue of processing large amounts of data in real time (128, 129), (ii) may provide scalable, cost-efficient data analytics solutions (130). Cloud computing, however, brings some technical difficulties, such as the ones related to high-throughput data transfer infrastructures, distributed computer power over very large non-parallelizable tasks and perhaps the main challenge (that we will discuss more in depth in a forthcoming section) which lies in adapting the current distributed storage and processing paradigms in big data, while simultaneously allowing for full confidentiality of the data (since some of it may be highly sensible in nature) (131).

However, a number of cloud computing resources are becoming a standard for several omic studies, as it can be exemplified by Basespace a cloud-based sequencing analysis environment by Illumina, by the EasyGenomics platform of the Beijing Genomics Institute (BGI) and by European-based Embassy clouds as part of the Elixir Collaboration, by the NGScloud2 over Amazon Web Services (AWS) or by Galaxy-Kubernetes integrated workflows to name but a few instances (132–139).

It is worth noticing that standard cloud computing designs using distributed systems, grid computing, parallel programming, and virtualization on top of multi-layered environments (134, 140) are becoming adopted in LSDA in precision medicine due to their applications in the development of robust and secure distributed analysis (132). Indeed, as we already mentioned, cloud computing in LSDA may be implemented under several paradigms, such as: Platform as a Service (PAAS) (141–143), Infrastructure as a Service (IAAS) (144, 145), and Software as a Service (SAAS) (35, 146, 147).

These different standards for cloud computing have their particular pros and cons when applied to LSDA in Precision Medicine; for instance PAAS designs are suited for in-house software development or to integrate already designed libraries that can be implemented either by the user or by the cloud provider. Here we can mention healthcare, biomedicine, and bioinformatics services by providers, such as Google App Engine, Microsoft Azure MapReduce Hadoop, and others. In contrast, IAAS providers commonly offer high performance computing and massive storage facilities (sometimes called *HPC-farms* or *data centers*) including only the minimum operating system/computing environment requirements: this is often the case of general plans offered by companies, such as Amazon Web Services, HP Cloud, Rackspace, and Joyent (148–151).

Of these different paradigms, SAAS results as the more complete, as well as the more costly and less flexible. In SAAS the user is able to perform LSDA via pre-established (sometimes customized) applications sitting on a remote cloud infrastructure. This provides almost immediate access and usability with minimum installation and customization requirements from the user. However, due to these very reasons, the user has less control over the specifics of both, the computing environment and the actual algorithms used to perform analysis. The risk is that some of the more sophisticated methods will develop into *black boxes*. A somewhat intermediate solution is what can be called *Code-as-a-service* that is, SAAS with full access to the code (often only by specific requirement of the user). This is the case of the Cloud BioLinux service (152). The Cloud BioLinux suite has a set of pre-installed services, like a Galaxy server (153), access to the BioPerl programming language (154), BLAST (155), R/Bioconductor (156), Glimmer (157), ClustalW (158), and other general purpose (mostly bioinformatic-related) libraries/packages/environments (35, 159, 160).

Aside from molecular biology and genomics oriented applications, SAAS has also been developed in areas, such as medical diagnostics. In this regard, one can mention *DXplain*, one of the earliest developed decision support systems available. DXplain that was created by scientists, physicians, and software engineers at Massachusetts General Hospital http://www.mghlcs.org/projects/dxplain. DXplain may be used as a search engine (akin to a searchable eBook) providing the concise yet detailed description of more than 2,600 medical conditions, indexed by their main signs and symptoms, as well as their etiology, pathology, and prognosis. More relevant to this discussion is the use of DXplain as a case analytics tool, processing a set of clinical findings (signs, symptoms, laboratory data) as an input to a computational intelligence engine that computes a ranked list of diagnoses related to the given clinical manifestations. Furthermore, DXplain provides supports its suggestions with evidence sources, suggests what further clinical information would be useful to collect for the conditions under consideration, and displays a list of relevant clinical manifestations (161, 162). IBM's *Watson Health* constitutes another example of a (commercial) SAAS system aimed to support clinical decision making by the use of computational intelligence methods www.ibm.com/watson-health/ (163). However, many researchers and clinicians have become skeptical of the tool due to initial over-promises from the company (164). Many other diagnostic support applications have been developed, most of them aimed at commercial use such is the case of *ISABEL* https://www.isabelhealthcare.com/ (165, 166) and others. However, due to commercial restrictions, their AI/ML assessment and their use in LSDA has been rather restricted (167, 168).

In the end, each health/biomedical/clinical research team will have to make a choice between these different levels of cloud services depending on its availability of technical staff (computational biologists, data scientists, statisticians, bioinformaticians, software engineers, and so on), the computer literacy and involvement of the biomedical researchers and the clinicians, the scope and extension of the projects and other constraints, including financial issues, local infrastructure, and confidentiality matters (169–173).

It is also needed to take into account that some LSDA applications in health and biomedicine demand usually high computing resources. One alternative that is gaining relevance recently is the design of hybrid servers combining traditional CPUs with Graphical Processing Units (GPUs). The use of GPUs on cloud-based environments is indeed favored, given their massively parallel architechture (MPA). MPA results advantageous not only for actual computations, but also for input/output (I/O) operations (174). An important fraction of GPU-based applications in computational biology and biomedicine are implemented under (175–177). However, it remains a challenging endeavor to develop and implement parallelization algorithms, efficient enough to make sense of heterogeneous data sources, such as the ones coming from omic technologies, from EHRs, population surveys (127, 178).

Aside from the already mentioned cloud-based solutions, most research and clinical institutions will need to build some local infrastructure and algorithmics suited for their particular needs. In the search for semi-automation and reproducibility, some relevant general tasks are better managed by resorting to specialized software and algorithmic suites developed with building workflows and pipelines in mind. We will present some of the more widely used of such suites or packages for LSDA useful in Precision Medicine in the following subsection.

### 4.2. Software Resources for Computational Medicine

Whether implementing local, cloud-computing, or hybrid solutions, choices need to be made regarding appropriate algorithms and software for data pre-processing, processing, and analytics. A number of general purpose approaches have been developed, such is the case of the suite of R-based algorithms and programs in the Bioconductor repositories (156), the pipeline management tools, such as Snakemake (179, 180) and Taverna (181) or the cloud-based development suites Helastic (182) and BioNimbus (183).

For sequence analytics, a central player for quite some time has been the genome analysis toolkit (GATK) by the Broad Institute (184, 185). The GATK suite has been developed for LSDA of genome sequencing data mainly focused on high-accuracy variant discovery and genotyping useful in the clinical and biomedical research environments (186). Other computational omic analysis tools useful in the context of Precision Medicine include dRanger for the automatic identification of somatic rearrangements in Cancer (187), Athlates for the determination of HLA immuno-genotypes from exome sequencing data (188), the Trinity suite for De Novo RNA-Seq analysis (189), the Hail library for scalable (bio-bank scale) genomic data exploration (190), and the GWAS analysis suite Plink (191), to name but a handful instances.

More broadly applicable suites have been also developed, such as GenePattern (192, 193), the running/development platform Galaxy (153, 194, 195). Biological function databases like Gene Ontology (196, 197) and its generalizations (198, 199), the MONA (multi-level ontology analyses) programs (200), and other medium-to-high level analysis tools, such as the network analysis suite Cytoscape (201) or the structural biology libraries BioDAS (202) to mention but a handful of the many available options.

Aside from genomics and purely molecular/omic studies, other computational tools have been developed and widely used in the biomedical and clinical settings. Such is the case of CellProfiler for image analysis and processing (203) that has been proved to be quite useful for machine learning applications (204, 205). Automating data throughput in biomedical and clinical applications may also be useful even for relatively low demand tasks under certain circumstances; for example, automated RT-PCR data processing as implemented in ARPA (Automated RT-PCR analysis) turned out to be crucial for testing efforts during the COVID-19 pandemic (206). AI/ML modeling based on facilitated access data may indeed become a key tool to tackle with current and future pandemics (207).

Moving on to clinical applications, some of the most popular computational tools for managing clinical data (particularly with clinical trials in view) are OpenClinica (208), the Integrated Data Repository Toolkit IDRT (209) and the VISTA trials suite (210), and the comorbidity risk assessment tool comoR (211). Tools for the management of high-throughput day-to-day clinical records commercial and academic/open source have flourished in recent times. Some of the more widely adopted open source software solutions are OpenEMR (212), OpenMRS (213), WorldVistA (214). Some of these tools are actually enabling capacities to allow for the implementation of data mining and computational learning on their databases (54), however, as previously discussed, caution must be taken when using EHR data for automated discovery since a number of potential biases and confounders may arise (215, 216).

There are also some R-packages useful to manage EHR data. Such is the case of EHR: an Electronic Health Record and Data Processing and Analysis Tool https://cran.r-project.org/web/packages/EHR/index.html (217, 218), as well as rEHRhttps://github.com/rOpenHealth/rEHR (219).

Other software solutions from the R ecosystem useful in the LSDA applications in the clinical practice include babsim.hospital, a hospital resource planner and simulator https://cran.r-project.org/web/packages/babsim.hospital/index.html (220); bp a blood pressure analytics tool https://cran.r-project.org/web/packages/bp/index.html; and card a toolkit to evaluate the autonomic regulation of cardiovascular physiology via integrating electrocardiography, circadian rhythms, and the clinical risk of autonomic dysfunction on cardiovascular health data https://cran.r-project.org/web/packages/card/index.html (221).

Other software packages include radtools a set of utilities to extract and analyze medical image metadata https://cran.r-project.org/src/contrib/Archive/radtools/ (222); psrwe a library useful to incorporate real-world evidence (RWE) into regulatory and health care decision making https://cran.r-project.org/web/packages/psrwe/index.html (223, 224); clinDataReviewhttps://cran.r-project.org/web/packages/clinDataReview/index.html an environment to support exploratory analysis of data in clinical trial settings, patientProfilesVis a tool to create patient profile visualizations for exploration, diagnostic or monitoring purposes during a clinical trial https://cran.r-project.org/web/packages/patientProfilesVis/index.html; and even healthyR a full suite to review common administrative hospital data. Although this latter application does not seem to be related to LSDA in Precision Medicine, it is not uncommon the application of AI/ML methods to administrative data to infer, for instance, social determinants of health.

## 5. Data Integration: Current Challenges

Computational limitations in LSDA for Precision Medicine are gradually being overcome. Deeper challenges, however, arise when we consider the question of how to develop coherent ways to *make sense of the data*, that is how to build models and analytical frameworks that allow biomedical scientists and clinicians to use all these currently available data types and resources in the best possible way as diagnostic and prognostic tools (225). In the context of genomics (and other omics) in biomedicine, important international efforts along these lines have been developed, such is the case of the ELIXIR-EXCELERATE collaboration (136), the STATegra project (226, 227), the SeqAhead consortium (228), and others (229, 230).

It must be stressed that most of the efforts of these—extremely relevant—endeavors are directed toward the integration of information on the *molecular* side of the spectrum of biomedical related data. Data integration at this level provides mathematical and relational models able to give a mechanistic description of the interplay between the molecular components of the cells (225, 231). This is of course fundamental to understand the rise of cellular and tissular phenotypes from its biochemical origins, but may result insufficient to account for the rise of disease in organs, individuals, and even populations. Recent advances have been done to extend these efforts to encompass LSDA on biological databases incorporating individual EHR data (232), as well as social and environmental information [the so-called social determinants of health (233)]; perhaps even incorporating constraints representing healthcare policy within a precision medicine framework (93, 234). Advances in AI will surely play a central role in the development of such integrated frameworks (235).

In this context, data integration allows the use of multiple data sources with several different (eve disparate) *pieces of evidence* to build (hopefully) interpretable models of the systems under study (236). Since these broad array of data sources may have quite different structures, levels of granularity and, in the case of quantitative measurements, different distributions and dynamic ranges, data integration is indeed a demanding endeavor, briefly subsumed in the question *how can we put together these data sources to improve knowledge discovery?* (237). Hence, being able to perform complex queries, build heterogeneous models and develop hierarchically nested data retrieval operations on multiple databases are core goals for data integration strategies useful for AI/ML models in Precision Medicine (235, 238–241).

LSDA in Precision Medicine is driven by two major sets of goals. On the one hand, we aim to develop *high level intuition* (HLE) from inductive analyses, via statistical learning and causal inference techniques. HLE may serve to sketch guidelines for current and future experimental and clinical research (242). On the other hand, AI/ML approaches may be useful for *automated reasoning* (AR), i.e., the non-supervised or semisupervised extraction of non-trivial patterns in *dynamic* databases (243–245).

### 5.1. The Need for Guidelines and Standardization to Support Precision Medicine

Machine learning and artificial intelligence approaches able to live up to these envisioned objectives will depend on the underlying data resources to a great extent. We will need, not only high throughput carefully curated databases, but also inter-operable data strategies. By creating integrated/integrable databases related to Precision Medicine we will enhance our *data discovery* and *data exploitation* capabilities, refine our algorithms for *statistical assessment of data-driven discovery* and improve our *data standardization*. Regarding data standards, there have been some advancements from the early days of the MIAME requirements (246, 247) for genomic data formats, now updated for next generation sequencing data (248) and even for single cell RNASeq experiments (249); to some more recent efforts for meta-data standardization (250, 251).

Focused efforts toward data standardization with AI/ML approaches in mind have been recently advanced. For instance, a multi-institutional group has recently compiled a document establishing guidelines on *Minimum information about clinical artificial intelligence modeling* by means of the MI-CLAIM checklist (252). MI-CLAIM has been developed as a tool to make reporting of AI/ML algorithms in medicine more transparent. This approach looks to solve issues related to interpretability, opaque documentation and scope of AI/ML methods in medicine. It consists of six parts: (i) Study design, (ii) Separation of data into partitions for model training and testing, (iii) Optimization and final model selection, (iv) Performance evaluation, (v) Model examination and (vi) Reproducible pipeline. Central to this standard is the MI-CLAIM checklist [Table 1 in (252)].

Aside from methods, standards need to be developed for all different aspects involved in biomedical data analytics and computational intelligence. From the patients/subjects to the clinical and analytical research, to academic and industrial approaches and back to the patients and clinicians. The National Patient-Centered Clinical Research Network (PCORNET) initiative https://pcornet.org/ of the US has been developed as *a national resource where health data, research expertise, and patient insights are available to deliver fast, trustworthy answers that advance health outcomes* (253). PCORNET was designed as a distributed data research network (DRN) built to facilitate multi-site observational and interventional research across the diverse (existent-at-the -time and future) clinical data research networks and other relevant players in the health data ecosystem.

By standardizing procedures, formats and approaches PCORNET looks up to deliver greater sample size and power of the studies, the ability to analyze the effects of the differences in practice and assessing heterogeneity in treatments and populations. It included the creation of a Data Standards Security and Network Infrastructure (DSSNI) task force aimed to identify the minimal data standards and technical specifications for data to be effectively shared and disseminated effectively. These actions will be directed to optimize the evaluation and improving quality assessment of the research projects and to maximize their concurrent impact (254). Other task forces within PCORNET are devoted to issues, such as Governance, Data privacy, Ethics, and regulation, Health system interactions, Patient and consumer engagement, Patient-generated outcomes, Clinical trials, Rare diseases, Biorepositories, and Obesity. These task forces (and other that are being added as they develop) are supervised by PCORNET's Project Management Office operating under a network-like structure rather than as a traditional hierarchical organization. The development and functioning of the approach are subject to continuous assessment and evaluation via the Foundational Data Quality model founded on the premises of optimal data curation (255).

A related initiative put forward by the National Center for Biomedical Computing of the US is the I2B2 (Informatics for Integrating Biology and the Bedside) https://www.i2b2.org/index.html. I2B2 was developed with the aim of *enabling effective collaboration for precision medicine, through the sharing, integration, standardization, and analysis of heterogeneous data from healthcare and research; through engagement and mobilization of a life sciences-focused open-source, open-data community*.. I2B2 was created as part of the NIH roadmap to advance precision medicine to provide the community of clinical investigators with a toolbox to integrate medical records, clinical data, and genomic technologies all at once (256). One of the foundations of I2B2's approach to data interoperability is data-model harmonization based on ontological representations, particularly those facilitating the involvement of subjects/patients and clinicians aside from biomedical researchers (257). The extent of influence of these actions is designed to further improve the way subjects are enrolled and followed-up in research study protocols, clinical trials and observational cohorts (258).

Ontologies are useful to provide a conceptual framework. In the case of automated and semi-automated data mining methods in biomedicine it is desirable to have a *standardized language*, easily translated into machine-readable text. This is precisely the aim of the *Biological Expression Language* (BEL). BEL is presented as *a language for representing scientific findings in the life sciences in a computable form. BEL is designed to represent scientific findings by capturing causal and correlative relationships in context, where context can include information about the biological and experimental system in which the relationships were observed, the supporting publications cited and the process of curation* https://bel.bio/. The elementary elements of BEL are known as BEL-assertions that are built as intermediate steps connecting natural language (as presented in say, academic writing or medical records) into machine-readable expressions. Such expression will then be *computable* with applications in tasks, such as logical modeling in database learning, systems biology verification studies or next generation EBM to name a few (259–261). Implementing language standards, such as BEL may prove beneficial, since it has been shown, for instance, that different approaches to process clinical notes using natural language analytics substantially affects the performance of predictive models in intensive care settings (262).

The biomedical data ecosystem is turning so complex that new standards are needed even to define what we call *evidence*. The large amounts of seemingly anecdotal data that are being produced nowadays have brought to attention issues like the so-called *real world evidence* (RWE). RWE refers to *data regarding the use, or the potential benefits or risks, of a drug derived from sources other than randomized clinical trials* (263). Large sampling spaces are behind RWE move from anecdotal to referential. However, not all the real world information should be treated as RWE. In this regard, there is a growing need for methods to assess when are these data sources rigorous and trustworthy enough as to be useful as a guideline or to be considered actual *evidence*. These issues result particularly relevant toward the definition of clinical pipelines in *digital therapeutics* (loosely defined as evidence based therapeutics basedon software applications to prevent, manage or treat a disease or medical condition) (264), often related with data obtained from wearables and other subject-based sources.

Data standardization is becoming central not only in the medical research, and personalized clinical practice settings. It has been recently discussed how clinical trial data sharing is essential for reproducibility of the findings, for visibility of the results, to improve subsequent trails or advanced clinical trial stages, to perform digital comparisons of effectiveness (which are much faster and cheaper than their traditional counterparts); but also to speed results reporting, to enable continuous learning and even to support the emergence of startups or enterprise ventures, among other issues (265). In order for shared data to be optimally usable, there is an obvious need for standardization.

Data is, of course, not the only issue that needs to be assessed and validated toward the widespread implementation of AI/ML approaches in Precision Medicine. Eaneff and coworkers have recently argued for the need of *algorithmic stewardship* for AI/ML technologies in the medical setting. In this regard, an algorithmic steward would be a person or group within a healthcare or biomedical research institution responsible for tasks, such as creating and maintaining an algorithmic inventory of the methods used in the institution, monitoring ongoing clinical use and performance of such computational tools, evaluating the safety efficacy and fairness of the methods and so on (266).

Data and methods constitute the most visible items within the biomedical analytics ecosystem; metadata, is however, progressively gaining a more relevant role for AI/ML in Precision Medicine, as it contains, in many cases, hints for the automated *labeling* or classification (even if approximate) tasks that will be further improved by the use of computational intelligence and statistical learning approaches (87, 267). We will further discuss this issue in the next subsection.

### 5.2. An Ocean of Metadata

Metadata has become a central player in contemporary LSDA endeavors in many fields, including biomedicine; particularly relevant for AI/ML approaches. For this reason, aiming for high quality, well-formatted and standardized metadata has become quite relevant (268). Indeed, a number of biomedical data analysis teams and consortia are encouraging the use of standardized metadata guidelines, exemplified, for instance by a *checklist* of relevant issues to consider when building and publishing companion metadata (250, 269, 270); since such metadata could be instrumental to implement data analytics, as well as AI/ML toward a precision medicine approach (267, 271).

Metadata may result also quite useful to enhance the statistical analysis, probabilistic models and training of learning machines. Using metadata to generate best priors may improve the outcomes of query optimization by resampling and bootstrapping (272–274), regularization of sparse datasets (275), as well as auxiliary source for multi-variate Bayesian analysis (200, 276, 277), multi-dimensional analyses on datasets with disparate dynamic ranges (278–281) among other instances (282–286).

Integrating multiple data and metadata sources takes even further the need to design, develop, and implement analysis algorithms able to handle heterogeneous data in the presence of noise accumulation, spurious correlations and incidental endogeneity, keeping a balance between statistical accuracy, computational efficiency, and interpretability (287–289).

LSDA approaches must be developed having in mind the presence of spurious correlations among unrelated covariates, challenging statistical inference by creating false positive findings (290). Incidental endogeneity occurs when a number of unrelated covariates become *correlated* via random correlations of their residual noises. A statistical approach to overcome some of these issues is the development of novel regularization methodologies (291–293) but also the use of outside cross-validation via independence screening tests (294, 295) that may be precluded by data unavailability from independent sources.

Taking these issues into account may require new models to implement metadata reporting standards (296, 297). Standardizing the way metadata is reported and retrieved in the biomedical and clinical settings will result critical for the development of generalistic machine learning approaches that make full use of these uniform data structures (298–300). It has been recently discussed that ignoring or bypassing such standards may jeopardize full research projects (301–303).

## 6. Ethical and Legal Challenges for Computational Medicine

Aside from the methodologic and logistic issues already discussed, integrating data sources aiming at LSDA in the context of Precison Medicine also brings out concerns related to the ethical and legal problems that may arise, for instance related to privacy and confidentiality. Regarding the purely technological aspects of this problem, most of the members of the community of data analyst in healthcare and biomedicine are actually confident that these can be solved with security and encryption approaches already used to protect personal financial data (6, 46, 304). Aside from privacy concerns, managing sensitive data implies having several layers of access to the data. This is so since *some* sensitive personal data may be extremely useful for population level studies needed to develop personalized medicine. However, even if it is unlikely that full disclosure of sensitive biomedical and clinical information is needed, there is a fraction—that need to be determined and agreed-upon in advance—of potentially sensitive information that results fundamental for the development of personalized medicine, not just for the individual in particular but also population and sub-population-wise (305).

Then a conundrum arises as how to accommodate smooth clinical and biomedical data widespread with efficient privacy practices. The goal here is to implement stringent rules that maximize data yield while preserving anonymity and data protection. Data specialists have proposed several strategies to accomplish this goal. Currently one of the most favored is centered in *mining designs* based on the so-called *minimally-invasive queries* (MIQs) designed ex-profeso to preclude (and in due case disclose/document) any abuse of sensitive data (306). In some sense MIQ approaches mimic and extend the practices that have been long held by the international health insurance community while dealing with privacy in the EHRs via guidelines, such as the *Health Insurance Portability and Accountability Act* (HIPPA). Aside from its enormous legal and bioethical consequences, HIPPA adoption induced the development of data protocols in biomedical informatics that will result useful—even if as a starting point—for the LSDA under the Precision Medicine paradigm. Full implementation of optimized data usage/protection protocols is still underway, however, important advances have been made (307–310).

Reaching an optimal balance between information protection and efficient data mining outputs presents itself as a complex endeavor: some experts from the biomedical ethics community advocate for a careful case-by-case analysis, though admittedly this will be too complex to be implemented in general purpose LSDA workflows. As an alternative to this it has been suggested that multi-level data encryption (311, 312) can be applied in such a way that only authorized personnel will have the decoding keys to have access of the different levels of information (313).

In order to lessen the burden of encryption, encryption must be selective so that only personal identifiers and other private features (that may help disclose such identifiers) should be encrypted. Quasi-identifiers (QIDs), such as location, ethnic profiling, age and employment information, and highly-specific genomic data may be subject to certain low-level encryption by following *differential privacy* standards (314, 315). Some caution needs still to be taken since individual QIDs may not be informative enough to disclose identity, but there may be mining-integration procedures that may be able to do so by arranging coupled queries as it has been already discussed in the context of large scale genomic and transcriptomic studies (316–318).

Aside from genomic sources, other data types that may be used as potential QIDs in the context of biomedical informatics include, for instance, photographs: it has been discussed that from image (and imaging) data, AI approaches are able to infer *barcodes* from cranial and facial morphological features, skin pigmentation, eye color, retina patterns, iris structure, as well as hair type and color (108, 317, 319–322).

These are but a handful examples of how biomedical and clinical data features may turn into QIDs potentially posing ethical dilemmas to LSDA in the context of Precision Medicine. In this context and with the advent of powerful AI/ML approaches, a question arises as to which queries are *valid* and which ones are not from the standpoint of ethics, privacy and confidentiality. It is expected that as AI/ML methods become more powerful, methodological adjustments should evolve to balance safety and non-triviality of the queries with the impact of the analyses. This call for an organized implementation of such features via standardized *query tools* compliant with the agreed (potentially also evolving) ethical standards of the community (313). This translates into further challenges for the computational tools for data mining and analysis that may be designed with hierarchical multi-layered data structures in mind from the start.

Protected health information (PHI) is a relevant issue in this regard since it potentially allow for individual identification. Developing methods to effectively de-identify sensible data, such as the one included in free-text clinical notes may become part of the solution to the ethical challenges of high throughput data mining in the clinical and biomedical settings. With this in mind, Norgeot and collaborators developed a customizable open source de-identification software called Philter (323). Philter https://github.com/BCHSI/philter-ucsf has shown to outperform well-known methods, such as the ones in the Physionet https://www.PhysioNet.org/physiotools/deid/ and Scrubber https://scrubber.nlm.nih.gov/files/ suites. Subject de-identification in clinical notes and similar documents since such corpora often contain detailed information about the state of individual patients, the evolution of their disease conditions, specific theraputics and outcomes. That kind of information that will result key for the development of Precision Medicine, but at the same time may pose privacy challenges unless effective de-identified.

In view of the advances in AI/ML and the ethical challenges that come as a consequence of these advances, design changes are needed not only in the analytics. Research protocols, clinical trials and documented medical procedures, for instance, must be revised since the personal decision to share or not personal healthcare information or participating in large scale biomedical research cohorts may change at the light of AI/ML advances. Hence, informed consent procedures may need to be adapted. This implies reframing the current paradigm for the protection of individual privacy and adopting ways to educate patients/participants on how the data collected may affect them and the what extent their data can or cannot be protected, contextualising this in terms of the potential benefits for them and for others (317).

It has been discussed that *re-educating* about the way they view their own data also implies increasing their involvement with how their data may be used to affect them and others. Indeed, one of the central tenets of Personalized Medicine is making healthcare, *personal*. In this regard, it is worth discussing the role that *data portability* will play in individual and collective decisions (324, 325). Integrating data analytics, privacy protection and data portability is, in brief, one of the current open problems in computational medicine and medical informatics (326–328).

Given all the twists and subtleties just discussed in the context of LSDA for Precision Medicine, it has been considered advantageous to document in all detail (or as comprehensively as possible given the particular context) how data is gathered, archived, processed, analyzed, disseminated, and used in each research study, clinical trial, or large-scale clinical follow-up. Guidelines have been currently advised as how to elaborate such a document termed a *data management plan* (DMP). We will briefly discuss on these matters in the next section.

## 7. The Importance of a Good Data Management Plan

In view of all the complexities associated with projects managing and analyzing large amounts of potentially sensitive data, writing down a comprehensive document with all the associated information, a data management plan document is considered advantageous (329–332). The purpose of the DMP is to establish guidelines about how the data will be treated during the course of the project and even what will happen after the project is finished. The DMP considers what will be done with the data from its collection, throughout the organization, pre-processing, and analysis stages. It considers data quality controls, database preservation, and documentation techniques used, as well as usage restrictions and conditions for the further use, dissemination and sharing, embargoes, and limitations.

The DMP document has been established to be compliant with the legal requirements for all involved institutions and funding agencies. It should specify what types of data are to be collected, the recommended (sometimes preferred, sometimes mandatory) formats to handle and preserve the data. It results relevant to mention the software requirements and computational resources used to store, process, analyze, and visualize the data. The expected volume and structure of the databases, as well as its sources, traceability and metadata information (329). The DMP must also mention the intended data preservation strategies, database organization (e.g., naming conventions, dictionaries, reports' systems, etc.), identification and de-identification procedures. It is also advisable to establish guidelines for database curators—in some cases, even for auditors—(for instance regarding data integrity, quality controls and data standardization). All these entries of the DMP must be compliant with normative and organizational principles detailed in the so-called *Project Data Policy* (PDP) section of the DMP. The PDP may include information on legal, administrative and even ethical restrictions to be considered when managing the data. In some cases, this has to make it extensive to associated software and metadata (331).

The data dissemination policy section of the DMP states how, when and whom will have access to the data and under what circumstances. It is recommended that a subsection assigning personal roles and responsibilities of the associated personnel is included to ensure good data governance. The DMP is, in brief a dynamic instrument that plays a normative role, but also serves as a registered account on the whole data workflows and procedures throughout the project. Hence, a good DMP contributes to a secure and smooth functioning of the whole LSDA project (333, 334).

## 8. Conclusions and Perspectives

Artificial Intelligence and Machine Learning (AI/ML) approaches have proven to be extremely relevant tools for the large scale analysis of biomedical and clinical data; central for the development of Personalized Medicine. Useful as they are, implementing AI/ML methods in the highly demanding medical applications, it is not an easy endeavor. A number of caveats, shortcomings and subtle points have to be taken into account (and in many cases, circumvented) in order to provide appropriate solutions for the individual and public health care to fully benefit from these emerging paradigms.

In this work, we have discussed about some of the central challenges, problems, and drawbacks found in the applications of the methods and designs of large scale data analytics within clinical and biomedical environments, in particular under a Precision Medicine perspective.

Some relevant points can be briefly summarized as follows:

- Precision Medicine has been recently presented as an emergent paradigm to approach healthcare in a more predictive, preventative, personalized, participatory way (sometimes also called P4 Medicine). Precision Medicine has strong ties with data intensive approaches, as well as with machine learning and artificial intelligence.
- To deliver the promise of Precision Medicine, computational learning approaches are to be nurtured by well-curated and nifty integrated data ecosystems.
- Data resources in the biomedical research, clinical and healthcare environments are becoming extremely large, and are complex, unstructured and heterogeneous, hence difficult to deal with individually, even more so to be integrated into a coherent framework.
- The universe of diverse data sources needs to be collected, pre-processed, processed, modeled, and integrated to construct such coherent frameworks useful for Precision Medicine (see Figure 1). This is much easier said than done.
- In order for machine learning models to give good results their input needs to be *good* data. Transforming existing data into optimized forms for AI/ML is essential.
- If medicine is to become *personalized*, we must embrace diversity, heterogeneity, biases, class imbalance, and other intrinsic features of *individuals*. There is a need to develop methodologies to rigorously operate under these constraints.
- To develop, implement, optimize, and improve on these methods, a number of challenges needs to be overcome. These include technical limitations, computational aspects (both software and hardware/infrastructure), mathematical and modeling issues, and even ethical, legal, and policy matters.
- We have presented and discussed some of these challenges, aiming at showing the state of the art in these different fields.
- We have introduced the need for data intensive endeavors, from the research arena to the clinical setting and the healthcare institution level to design and implement a data management plan to consider the issues that may arise and planning ahead for their solution.

We are convinced that the development and implementation of tailor-made (or at least well-customized) approaches, in terms of robust statistical and computational algorithms, supported by optimized frameworks for data acquisition, storage, management, and analytics, but also by well-integrated software solutions and guided by solid ethical policies compliant with a deep respect for privacy, confidentiality, and individuality; is an ambitious but attainable goal. Hence, by combining state of the art computational learning methods and techniques with the best data acquisition and management practices the promise of AI/ML in Personalized Medicine may be delivered.

## Author Contributions

EH-L conceived the project. EH-L and MM-G performed research and drafted the manuscript. All authors reviewed and approved the manuscript.

## Funding

This work was supported by CONACYT (grant no. 285544/2016 Ciencia Bsica, and grant no. 2115 Fronteras de la Ciencia), as well as by federal funding from the National Institute of Genomic Medicine (Mexico). Additional support has been granted by the National Laboratory of Complexity Sciences (grant no. 232647/2014 CONACYT). EH-L acknowledges additional support from the 2016 Marcos Moshinsky Fellowship in the Physical Sciences.

## Conflict of Interest

The authors declare that the research was conducted in the absence of any commercial or financial relationships that could be construed as a potential conflict of interest.

## Publisher's Note

All claims expressed in this article are solely those of the authors and do not necessarily represent those of their affiliated organizations, or those of the publisher, the editors and the reviewers. Any product that may be evaluated in this article, or claim that may be made by its manufacturer, is not guaranteed or endorsed by the publisher.
